# The causal role between circulating immune cells and diabetic nephropathy: a bidirectional Mendelian randomization with mediating insights

**DOI:** 10.1186/s13098-024-01386-w

**Published:** 2024-07-16

**Authors:** Ning Shen, Shangwei Lu, Zhijuan Kong, Ying Gao, Jinxiu Hu, Shuxuan Si, Junlin Wang, Jie Li, Wei Han, Rong Wang, Zhimei Lv

**Affiliations:** 1 Department of Nephrology, Shandong Provincial Hospital, Shandong University, Jinan, 250021 Shandong China; 2 Department of Nephrology, Shandong Provincial Hospital Affiliated to Shandong First Medical University, Jinan, 250021 Shandong China

**Keywords:** Immune cells, Diabetic nephropathy, Mendelian randomization, Metabolite

## Abstract

Diabetic nephropathy (DN) is a critical inflammatory condition linked to diabetes, affecting millions worldwide. This study employs Mendelian randomization (MR) to explore the causal relationship between immune cell signatures and DN, analyzing over 731 immune signatures and incorporating data from 1400 metabolites to investigate potential mediators. Despite no statistically significant influence of DN on immunophenotypes after FDR correction, some phenotypes with unadjusted low P-values warranted mention, including CD34 on Hematopoietic Stem Cell (Myeloid cell Panel), CD45 on CD33^−^ HLA DR^−^ (Myeloid cell Panel). Furthermore, three immunophenotypes were identified to have a significant impact on DN risk: CD16^−^CD56 on HLA DR^+^ NK (TBNK Panel), CD45 on HLA DR^+^ T cell (TBNK Panel), and CD33^dim^ HLA DR^+^ CD11b^+^ AC (Myeloid cell Panel). Our findings underscore the critical role of immune cells in DN, highlighting potential mediators and offering new insights into its underlying mechanisms.

**Supplementary Information:**

The online version contains supplementary material available at 10.1186/s13098-024-01386-w.

## Introduction

The International Diabetes Federation (IDF) latest statistical data shows that approximately 537 million adults (aged 20–79) worldwide had been diagnosed with diabetes in 2021 [1]. Diabetic nephropathy (DN) is a significant microvascular complication that is gaining increasing attention from the medical community and society at large [2]. With the dramatic increase in the number of people with diabetes, the kidneys, as vital target organs, are experiencing a growing incidence of damage. Approximately 40% of individuals with diabetes may develop DN [3]. Hypertrophy and thickening of the basement membrane, as well as the accumulation of extracellular matrix components, are the structural changes in the kidneys caused by DN. This ultimately leads to end-stage glomerular occlusion and tubulointerstitial fibrosis [4, 5].

Traditionally, the primary pathogenic mechanisms of DN are believed to involve metabolic disturbances, hemodynamic changes, oxidative stress, and so on [6]. In recent years, more and more evidence has indicated that chronic inflammatory reactions caused by immune cells are a vital factor in the pathogenesis of DN [7, 8]. Studies have indicated the widespread presence of various immune cells, including macrophages and T cells, in the glomeruli or interstitium of the kidneys in patients with DN. Their abnormal infiltration and activation within the renal interstitium are considered potential immunopathological mechanisms underlying DN damage [9]. Despite numerous studies investigating the essential mechanisms by which various immune cells play a role in the pathogenesis of DN, DN is a complex disease influenced by multiple factors, making it challenging to isolate the specific contributions of immune cells. While there is often observed activity of immune cells associated with DN, whether immune cell activation is a cause or a result of the disease may not always be clear. Additionally, the mechanisms by which various immune cells, including NK cells and mast cells, contribute to the onset of DN are not well-understood, requiring further research to confirm the potential mediators through which immune cells may influence the complex pathogenesis of DN.

Single nucleotide polymorphisms (SNPs) are not affected by postnatal factors (including exposure, confounding factors, and outcomes), unlike observational studies that struggle to determine the causal sequence between exposure and outcome [10, 11]. MR is a method of analyzing genetic variables that adheres to Mendel's laws of inheritance. The causal connection between observed exposure associations and clinically relevant outcomes is determined using SNPs as IVs [12, 13]. Therefore, to understand the causal link between immune cells and DN, we opted for MR, providing a more complete understanding of how immune cells contribute to the pathogenesis of DN.

This research aims to assess the causal link between 731 immune cell types and DN using bidirectional MR based on genomic data. Subsequently, to identify potential mediators linking immune cells and DN, a two-step MR method will be utilized.. The aim of this study is to provide valuable insights for preventing and treating DN.

## Materials and methods

### Study design

To investigate the causal connection between 731 immune cell features categorized into seven groups and DN, a two-sample MR analysis was performed. Furthermore, we utilized a two-step MR method to examine if it has possible mediators that connect immune cells to DN. Effective IVs must satisfy three key assumptions when using genetic variation as a representation for risk factors in MR studies. (1) Genetic variation and exposure have a direct correlation. (2) Genetic variation does not have any connection to potential confounding factors that may arise between exposure and outcomes. (3) The results through alternative pathways are not influenced by genetic variation, except for exposure [14, 15]. All the data used in our study come from rigorously reviewed GWAS datasets published in public databases and had been approved by the institutional review council for their studies.

### Genome-wide association study (GWAS) data sources for DN

We analyzed the data for MR from the Open GWAS database of the Integrative Epidemiology Unit (IEU), which was predominantly composed of publicly accessible GWAS summary datasets. Five European cohorts of 213,746 individuals (3283 DN cases and 210,463 controls) were included in a GWAS, where statistics of DN traits were obtained.

### Immunity-wide GWAS data sources

The immune cell GWAS data was obtained from a study on genetic characteristics of immune cells, which has accession numbers that range from GCST0001391 to GCST0002121 [16]. In this study, researchers used flow cytometry to analyze 389 median fluorescence intensities (MFI) that indicated surface antigen levels, 192 relative cell counts (RC), 118 absolute cell counts (AC), and 32 morphological parameters (MP). In addition, the researchers also used flow cytometry to analyze the immune cell data obtained from donors based on their cell phenotypes, dividing them into seven groups: TBNK panel, Treg panel, Matura stages of T-cell panel, DC panel, B-cell panel, Monocyte panel, and Myeloid cell panel. The original GWAS on immune traits was conducted on a population of 3757 Sardinians. Subsequently, approximately 22 million high-quality markers were retained for association analysis, wherein adjustments were made for gender, age, and age2 as covariates [17].

### GWAS data sources for metabolites

The GWAS Catalog, located at https://www.ebi.ac.uk/gwas/, was utilized to obtain the summary statistics for the GWAS. The accession numbers assigned to European GWASs ranged from GCST90199621 to GCST90201020, while the accession numbers assigned to non-European GWASs ranged from GCST90201021 to GCST90204063. This study encompassed a genome-wide association analysis of 1091 blood metabolites and 309 metabolite ratios, the research suggested that the discovery of associations between 690 molecules at 248 locations and 143 metabolite ratios at 69 locations. Furthermore, a careful analysis of metabolite genes and gene expression data resulted in the identification of 94 effector genes and 48 metabolite ratios linked to 109 metabolites [18].

### Selection of IVs

According to recent studies [16, 19], a statistical significance threshold of 1 × 10^−5^ was determined for IVs linked to each immune trait. Standardization of direction of effect ensured that SNP effects on each immune trait and DN were related to the same allele. In addition, our study excluded SNPs showing linkage disequilibrium (LD) from the results data set, LD needs to satisfy r^2^ < 0.001 and genetic distance within a 10 Mb window. We excluded IVs with low F statistics (F > 10) from our analysis to ensure their robustness. An F statistic greater than 10 is generally considered indicative of a strong instrument, reducing the risk of weak instrument bias. In addition, in order to meet the second condition of Mendel's randomization hypothesis, we also used the LDTrait tool to analyze confounding factors, and removed SNPs that may directly affect diabetes nephropathy. The same data processing steps were applied to the metabolite data. Additionally, we identified 21 IVs for DN for further MR analysis. The same data processing steps were applied to the metabolite data. We performed the same processing on the metabolite data. Additionally, we identified 21 IVs for DN for further MR analysis.

### Statistical analysis

The process of performing MR analysis was carried out through the use of R software (http://www.Rproject.org) and the 'Two-Sample MR' package (version 0.5.8) [20]. Various statistical methods were used to evaluate the causal connection between 731 immunophenotypes and DN, including IVW, MR‒PRESSO, weighted mode, MR-Egger, weighted median and simple mode [21–24]. The best way to use valid IVs is by utilizing the IVW analysis, which is widely acknowledged as the most efficient approach. The IVW estimate is consistently efficient and close to the true value when the genetic IVs do not have pleiotropic effects and the sample size is sufficient [25]. As a result, the IVW approach was selected as the main method for carrying out MR analysis. The heterogeneity among selected IVs was tested using Cochran's Q statistic and corresponding p values. The multiplicative random effects IVW method is used instead of the default fixed-effects IVW when there is heterogeneity in the selected IVs [21]. The utilization of MR-Egger, a method that is commonly utilized, was employed to test the impact of horizontal multiplicity. The significance of its intercept term indicates the presence of horizontal multiplicity [26]. Moreover, scatter plots and funnel plots were utilized in this study. The outcome was unaffected by any outliers as indicated by the scatter plots. Furthermore, the funnel plots were used to show how strong the correlation was and that there was no heterogeneity in this study. Moreover, this research also introduced a two-step MR approach for intermediary analysis, aiming to elucidate the potential existence of an intermediary pathway between immune cells and DN.

## Result

### The impact of DN on immunophenotypes

**Fig. 1:**
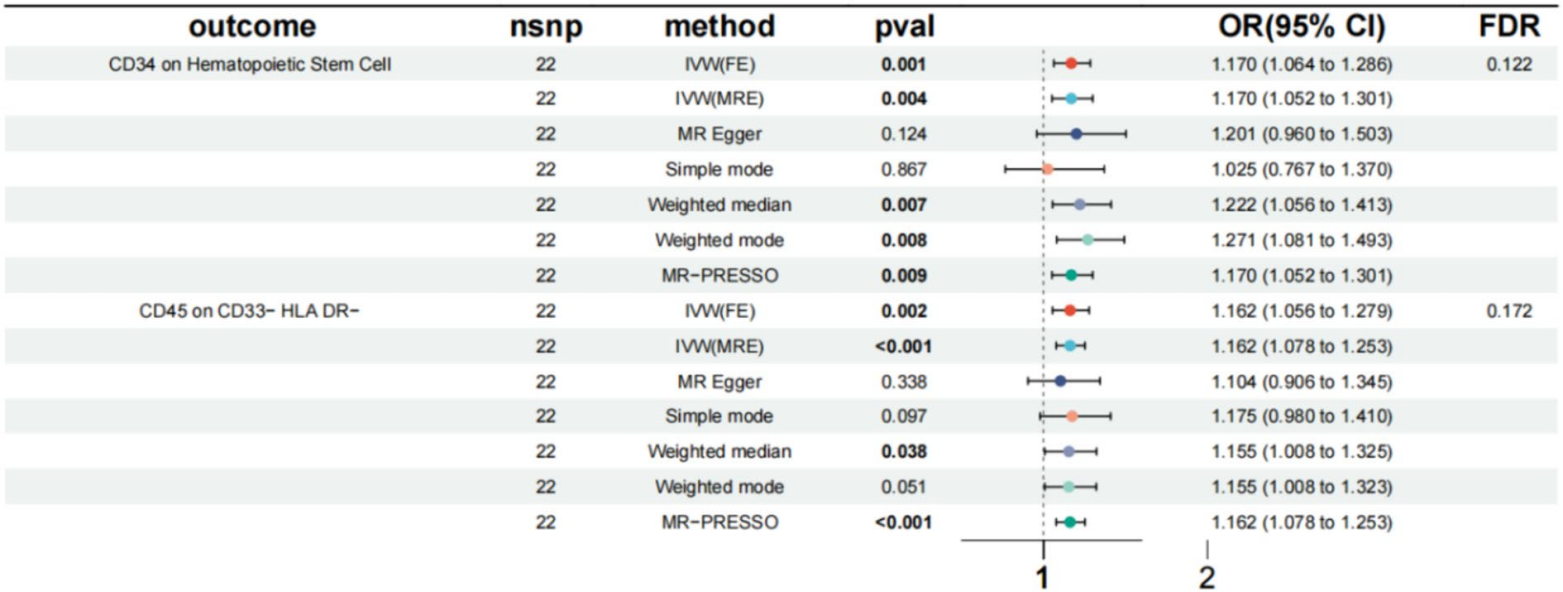
Forest plots showed the causal associations between DN and immune cell traits

DN's impact on immunophenotypes was determined through an MR analysis. Despite using the FDR method to adjust multiple tests, there were no immune traits that were significant at a level below 0.05. After applying the FDR adjustment with a significant level of less than 0.2, our investigation revealed that DN can cause an increase in CD34 on hematopoietic stem cells (Myeloid cell Panel) (OR = 1.170, 95% CI = 1.064 to 1.286, P = 0.001, P_FDR_ = 0.122) by using the IVW method. Three additional methods yielded similar outcomes. CD45 on CD33^−^ HLA DR^−^ (Myeloid cell Panel) was also found to be increased (OR = 1.162, 95%CI = 1.056 ~ 1.279, P = 0.002, P_FDR_ = 0.172). Similar results were observed by using weighted median and MR-PRESSO (Fig. 1 and Supplementary Table S1, S2).

In addition, the observed causal associations were confirmed to be robust through detailed information from the sensitivity analysis (Supplementary Table S1, S3). Meanwhile, we used two methods, including the intercept of MR Egger and the global test of MR-PRESSO, to examine the potential level of pleiotropy in the results, which proved that no level of pleiotropy was found in our study (Supplementary Table S1). Scatter plots and funnel plots also indicated the stability of the results (Supplementary Fig. S1, 2).

### The impact of immunophenotypes on DN

**Fig. 2:**
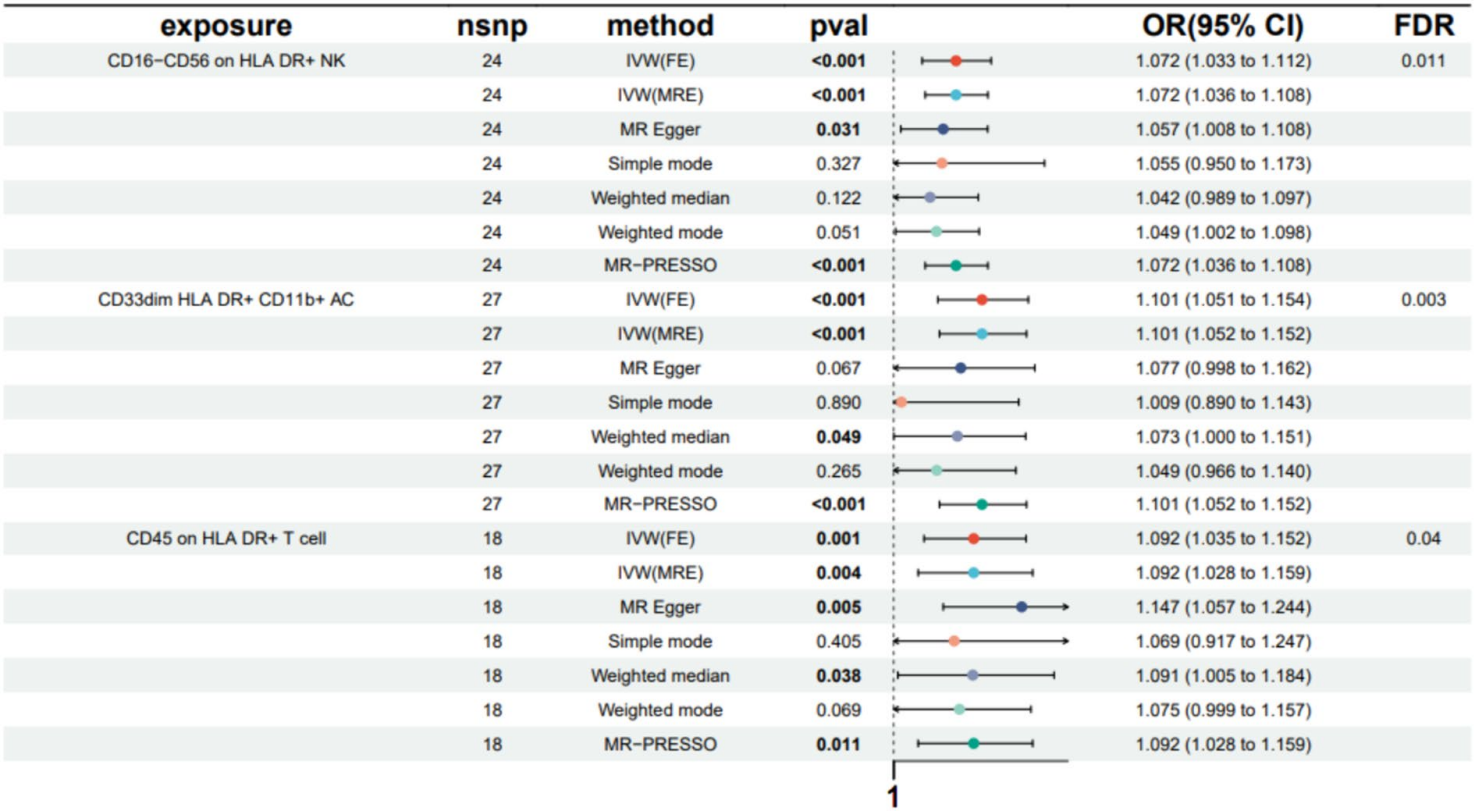
Forest plots showed the causal associations between immune cell traits and DN incidence

Three immunophenotypes had promoting effects on DN after FDR adjustment (P_FDR_ < 0.05): CD16^−^ CD56 on HLA DR^+^ NK (TBNK Panel), CD45 on HLA DR^+^ T cell (TBNK Panel), and CD33^dim^HLA DR^+^CD11b^+^AC (Myeloid cell Panel). Specifically, the odds ratio (OR) of CD16^−^CD56 on HLA DR^+^ NK on DN risk was estimated to be 1.072 by using the IVW method (95% CI = 1.033 ~ 1.112, P = 2.5 × 10^−4^, PFDR = 0.01), which was consistent with the MR Egger, Weighted Mode and MR-PRESSO. The OR of CD33^dim^ HLA DR^+^ CD11b^+^ AC on DN risk was estimated to be 1.101 (95% CI = 1.051 ~ 1.154, P = 5.84 × 10^−5^, P_FDR_ = 0.003) by using the IVW method. Using another method, similar results were observed: MR-PRESSO (OR = 1.101, 95% CI = 1.052 ~ 1.152, P = 3 × 10^–4^). The OR of CD45 on HLA DR^+^ T cell on DN risk was estimated to be 1.092 (95% CI = 1.035 ~ 1.152, P = 0.001, P_FDR_ = 0.033) by using the IVW method. Other methods resulted in similar findings (Fig. 2 and Supplementary Table S4).Moreover, neither the intercept of MR-Egger nor the global MR-PRESSO test showed any evidence of horizontal pleiotropy for either of these associations. The robustness of the observed causal associations was validated by the sensitivity analysis through the provision of detailed information (Supplementary Table S5). In addition, the results' stability was verified through scatter plots and funnel plots ( Supplementary Fig. S3).

### Exploration of potential mediators between immune cells and DN

**Fig. 3:**
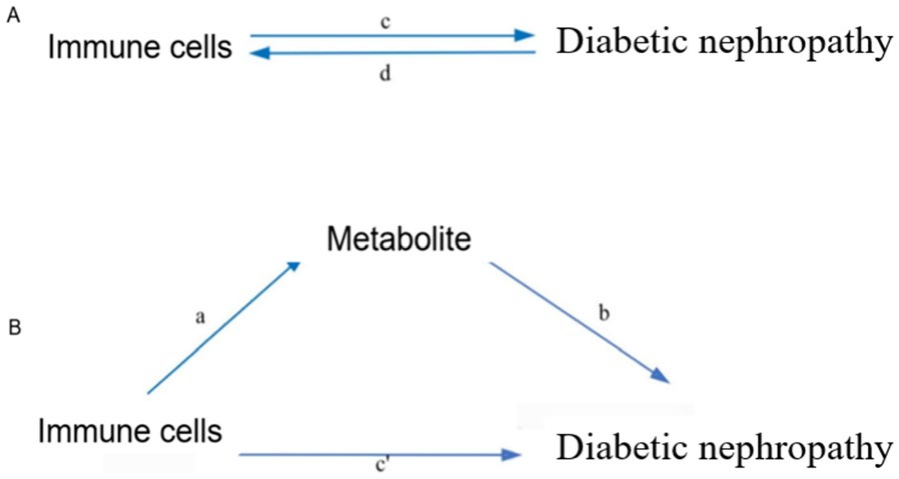
Diagrams illustrating associations examined in this study. **A** The total effect between immune cells and Diabetic nephropathy (DN).c is the total effect using genetically predicted immune cells as exposure and DN as outcome. d is the total effect using genetically predicted DN as exposure and immune cells as outcome. **B** The total effect was decomposed into: (i) indirect effect using a two-step approach (where a is the total effect of immune cells on Metabolite, and b is the effect of Metabolite on DN) and the product method (a × b) and (ii) direct effect (c′ = c – a × b). Proportion mediated was the indirect effect divided by the total effect

**Fig. 4:**
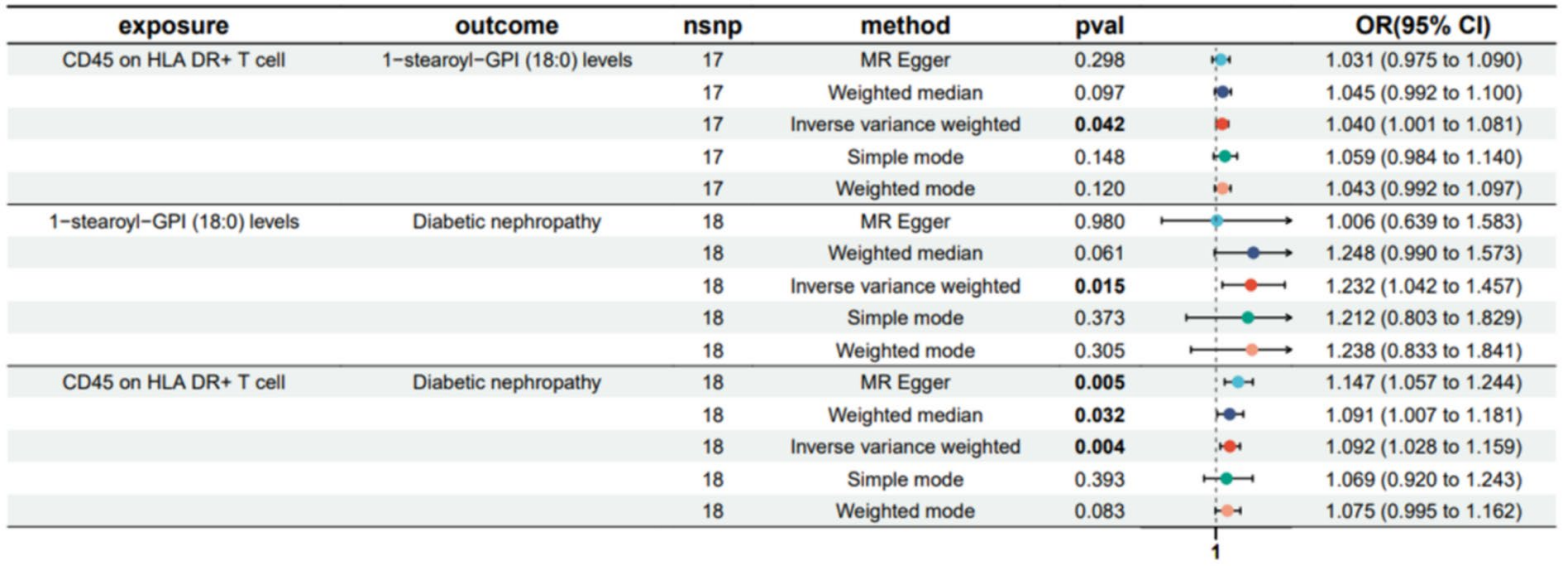
Forest plot to visualize the causal effects of 1-stearoyl-GPI (18:0) levels with CD45 on HLA DR^+^ T cell and Diabetic nephropathy (DN)

**Fig. 5:**
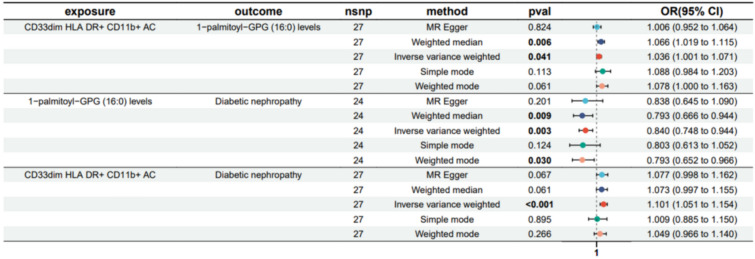
Forest plot to visualize the causal effects of 1-palmitoyl-GPG (16:0) levels with CD33^dim^ HLA DR^+^ CD11b^+^ AC and Diabetic nephropathy

**Fig. 6:**
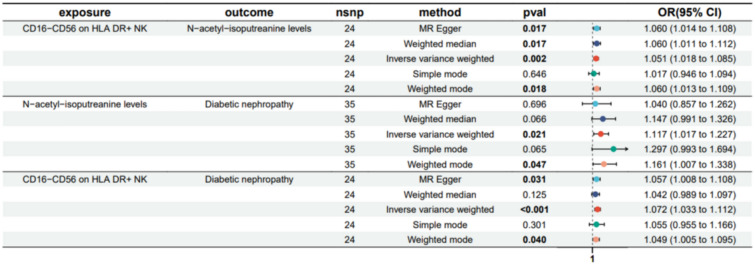
Forest plot to visualize the causal effects of N-acetyl-isoputreanine levels with CD16^−^CD56 on HLA DR^+^ NK and DN

Our research focused on analyzing mediation using a two-step MR design to explore potential causal pathways mediated by immune cells that can lead to diabetic nephropathy outcomes. The overall effect of immune cells on DN is divided into two parts: one of the ways immune cells affect DN is directly, and the other way is indirectly through mediators (Fig. 3). To make our analysis more comprehensive, we used additional statistical methods like MR-Egger regression, weighted median, simple mode, weighted mode, and MR-PRESSO. These methods were utilized to rigorously assess the quadratic relationship, thereby offering a sensitivity analysis to complement our IVW findings (Figs. 4, 5, 6 and Supplementary Table S6).

**Fig. 7:**
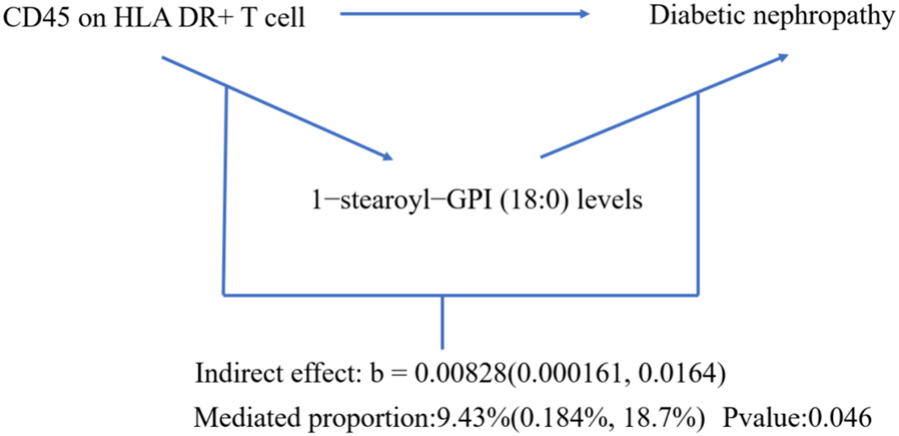
Schematic diagram of 1-stearoyl-GPI (18:0) levels mediation effect

**Fig. 8:**
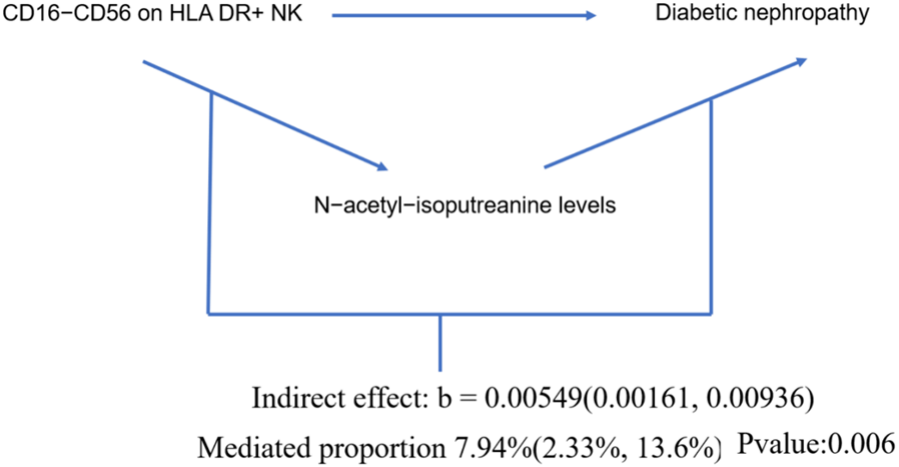
Schematic diagram of N-acetyl-isoputreanine levels mediation effect

**Fig. 9:**
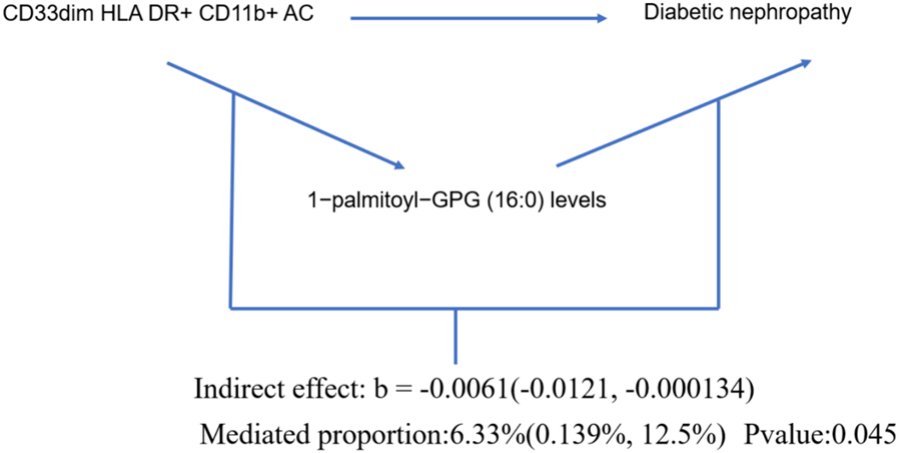
Schematic diagram of 1-palmitoyl-GPG (16:0) levels mediation effect

The result shows that the 1-stearoyl-GPI (18:0) levels were found to mediate CD45 on HLA DR^+^ T cell on Diabetic nephropathy and The N-acetyl-isoputreanine levels were found to mediate CD16^−^CD56 on HLA DR^+^ NK on DN (Figs. 7, 8). Moreover, the 1-palmitoyl-GPG (16:0) levels were found to have suppressing effect between CD33^dim^ HLA DR^+^ CD11b^+^ AC and DN, which means after reducing the 1-palmitoyl-GPG (16:0) levels, the force of the CD33^dim^ HLA DR^+^ CD11b^+^ AC on the DN will increase (Fig. 9).

## Discussion

DN is the main culprit of End-Stage Kidney Disease (ESKD) worldwide [27]. ESKD is a severe and complex condition with poor prognosis that has a significant impact on the quality of life and life expectancy of those affected, and it is associated with a high mortality rate. Current treatments for DN rely on multifactorial interventions, including lifestyle changes, and primarily target the simultaneous control of blood sugar, blood pressure, and lipid levels [28]. However, the clinical benefits of existing treatment strategies are limited, and many patients still progress to ESKD, highlighting the need for new treatment strategies.

An increasing body of research demonstrates the pivotal role of immune cells in the pathogenic mechanisms of DN. As far as we're aware, this is the very first MR analysis that focuses on the causal connections between different immune phenotypes and DN. According to this study, the CD33^dim^ HLA DR^+^ CD11b^+^ AC (Myeloid cell Panel), CD16^−^CD56 on HLA DR^+^ NK cell (TBNK Panel) and CD45 on HLA DR^+^ T cell (TBNK Panel) among four immune traits (MFI, RC, AC, and MP) have significant causal effects on DN (FDR < 0.05), which is important in exploring the pathogenesis of DN. Additionally, DN was found to have causal effects on the CD34 on hematopoietic stem cells and CD45 on CD33^−^ HLA DR^−^ (FDR < 0.2), potentially offer new directions for diagnosing DN and its subsequent complications in the future.

According to our study, a rise in the number of Myeloid-Derived Suppressor Cells (MDSCs) that express CD33^dim^ HLA DR^+^ CD11b^+^ is linked to the onset of DN. MDSCs have two representative subtypes: polymorphonuclear MDSCs have an immunophenotype of CD33dim, while monocytic MDSCs often express higher levels of CD33 [29]. Observational studies have indicated that polymorphonuclear MDSCs increase in Type 2 DN patients, and associated with kidney disease progression [30]. At present, Hua et al. found that HLA-DR expression results in myeloid cells with a phenotype and functional characteristics similar to fibroblasts [31]. Many studies also suggest a potential association between immune cells and renal fibrosis in DN. Combining our research results, it is speculated that CD33^dim^ HLA DR^+^ CD11b^+^ MDSCs may be related to renal fibrosis in the development of DN. Unfortunately, there is currently no specific research on CD33^dim^ HLA DR^+^ CD11b^+^ MDSCs.

Up to now, research on DN and immune cells has primarily focused on macrophages and T cells, with little knowledge about NK cells. It is noteworthy that we observed an increased level of CD56^+^CD16^−^HLA-DR^+^ NK cells, which is associated with an elevated incidence of DN. HLA-DR positive NK cells produce more IFN-γ and undergo more degranulation when exposed to various stimuli than HLA-DR-negative cells [32], which induces renal fibrosis by promoting collagen deposition [33]. Combining our research findings, we speculate that this could be a potential factor contributing to the progression of DN, which requires further exploration. NK cells expressing CD16^−^ CD56 primarily express granzyme A (GZMA), an inflammatory protease [34]. GZMA, as an inflammatory molecule, regulates the production of inflammatory cytokines such as IL-1β, TNFα, and IL6 [35], all associated with DN [33]. Elevated GZMA levels correlate with increased DN risk and reduced glomerular filtration rate [36], suggesting GZMA as a potential therapeutic target for DN, warranting further investigation.

Our research found that an increased number of HLA-DR^+^ T cells expressing CD45 is associated with the onset of DN. CD45, a type of protein called a receptor-type tyrosine phosphatase, is found abundantly in T cell membranes [37]. The Src family kinase Lck is induced by CD45, which results in phosphorylation of the T-cell antigen receptor complex, a pivotal component in the transduction of T-cell antigen receptor signals [38]. In fact, the crucial role of T cells in the onset of DN has been extensively studied. However, the role played by CD45 on HLA-DR T cells in DN onset remains ambiguous. We speculate that the downstream reaction activated by CD45 is an important target for T cells causing the onset of DN.

Through reverse MR, we have also discovered that the onset of DN leads to an increase in the quantities of two immune cell types: CD34 hematopoietic stem cells (HSC) (Myeloid cell Panel) and CD45 on CD33^−^HLA-DR^−^MDSCs (Myeloid cell Panel). Although we relaxed the significance threshold to 0.2 after FDR correction, it is noteworthy that these results already demonstrated strong statistical significance in the preliminary analysis before correction, suggesting their potential key role in the pathogenesis of DN.

Observational studies have previously found a significant reduction in the number of CD34^+^ HSC in diabetic nephropathy, with the microalbumin/creatinine ratio of patients moderately negatively correlated with the number of these cells [39]. This is inconsistent with the results obtained through MR. We speculate that the increase in CD34 on Hematopoietic Stem Cells may be related to the development of subsequent diseases in diabetic nephropathy, warranting further exploration.

It is noteworthy that Xian H et al. identified CD45 as a specific STAT3 phosphatase in MDSCs and further discovered that CD45 is a sumoylated protein [40]. The SUMO pathway regulates various cellular processes, activating NF-κB, TGF-β, MAPK, and inhibiting Nrf2 to exacerbate oxidative stress-induced DN [41]. SUMO1/sentrin-specific protease 1 (SENP1) can deconjugate SUMOylated CD45. Studies have found that SENP1 deficiency increases CD45 SUMOylation, and SENP1 inhibits the proliferation and function of MDSCs through the CD45-STAT3 signaling axis [40]. Whether SENP1 can slow down the progression of DN remains worth exploring, and our research provides new insights for treating DN.

To further explore how the three aforementioned immune cells contribute to DN, we employed a two-step MR to investigate their potential mediators. We identified mediators for each of the three immune cells, namely 1-palmitoyl-GPG (16:0) levels, 1-palmitoyl-GPG (16:0) levels, and N-acetyl-isoputreanine. Unfortunately, there is currently no reported research on the two metabolites, 1-palmitoyl-GPG (16:0) levels and 1-palmitoyl-GPG (16:0) levels. N-acetyl-isoputreanine is an amino acid that plays a role in polyamine metabolism and acts as the final product of this process [42]. Polyamines are essential biomolecules widely present in cellular metabolism. High glucose levels may cause abnormal polyamine metabolism in rat kidney tissues, inducing podocyte apoptosis and reduced autophagy, which could be a crucial mechanism in DN [43]. The content of polyamines and the glycolytic supply play a pivotal role in the immune activity of NK cells [44]. However, there is no related research on the toxic effects of polyamine metabolism end products on diabetic nephropathy. Our findings provide new insights into the pathogenesis of DN.

Our study's findings have significant clinical value. Personalized immunomonitoring using flow cytometry can aid in early detection and intervention for DN patients by monitoring CD33dim HLA DR + CD11b + AC, CD16-CD56 on HLA DR + NK cell and CD45 on HLA DR + T cell. This allows for early identification of abnormal immune responses and the development of tailored immunosuppressive treatments, such as anti-CD45 antibodies. Early intervention could slow DN progression and improve patient outcomes. Targeting specific immune cells, like reducing CD33dim HLA DR + CD11b + AC MDSCs to lower renal fibrosis risk and inhibiting granzyme A (GZMA) in NK cells to reduce inflammation, shows promise. Combining these therapies with standard treatments may enhance efficacy. Personalized treatment plans based on immunoprofiles can optimize outcomes and minimize side effects. These insights support the development of novel, individualized treatment strategies, with future large-scale studies needed to validate immune markers and integrate multi-omics data to uncover underlying mechanisms.

The findings of this research are derived from genetic IVs, and causal inference is accomplished through various magnetic resonance analysis methods. The outcomes are dependable and not subject to horizontal pleiotropy and other factors. Nevertheless, our research has limitations too. First, the assessment of horizontal pleiotropy is not complete even with multiple sensitivity analyses. Second, our inability to conduct more stratified analyses on the population was a result of the lack of individual information. Third, our study's findings cannot be generalized to other ethnicities because they are based on European databases, which limits the universality of our results. Fourth, the genetic predictions of DN mediated by the three identified mediators in our study are all below 10%, indicating a low genetic prediction accuracy. To quantify the role of other mediators, there is a need for more research.

## Conclusion

In summary, our comprehensive bidirectional MR analysis has revealed causal relationships between multiple immune phenotypes and DN, pointing out the complex interaction patterns between the immune system and DN. Our study may give researchers a new way to examine the biological mechanisms of DN, which could lead to more effective intervention and treatment. Furthermore, we used a two-step MR to examine the possible pathways that link immune cells and DN, with a small proportion of the effect mediated by some metabolites, but a majority of the effect of immunophenotypes on DN remains unclear. Further investigation into additional risk factors as potential mediators is required.

### Supplementary Information

Supplementary Material 1. Table S1: Causal effects of DN on immune cells.

Supplementary Material 2. Table S2: IVW results of the causal effect of DN on immune cells.

Supplementary Material 3. Table S3: Sensitivity analysis results of causal effects of DN on immune cells.

Supplementary Material 4. Table S4: Results of the causal effect of immune cells on DN.

Supplementary Material 5. Table S5: Sensitivity analysis results of causal effects of immune cells on DN.

Supplementary Material 6. Table S6: The Results of two-step MR.

Supplementary Material 7. Fig. S1: Causal effects of DN on immune cells.

Supplementary Material 8. Fig. S2: Causal effects of DN on immune cells.

Supplementary Material 9. Fig. S3. Causal associations between immune cells and DN.

## Data Availability

Data is provided within the manuscript or supplementary information files.
