# Supplementary material for: The causal role between circulating immune cells and diabetic nephropathy: a bidirectional Mendelian randomization with mediating insights
Source: Diabetol Metab Syndr. 2024 Jul 16;16:164. doi: 10.1186/s13098-024-01386-w (PMC11253417; doi:10.1186/s13098-024-01386-w)
Supplement: Supplementary file 7 — Supplementary Material 7. Fig. S1: Causal effects of DN on immune cells. [file 13098_2024_1386_MOESM7_ESM.docx]

**Supplementary Fig. S1 Causal effects of DN on immune cells**


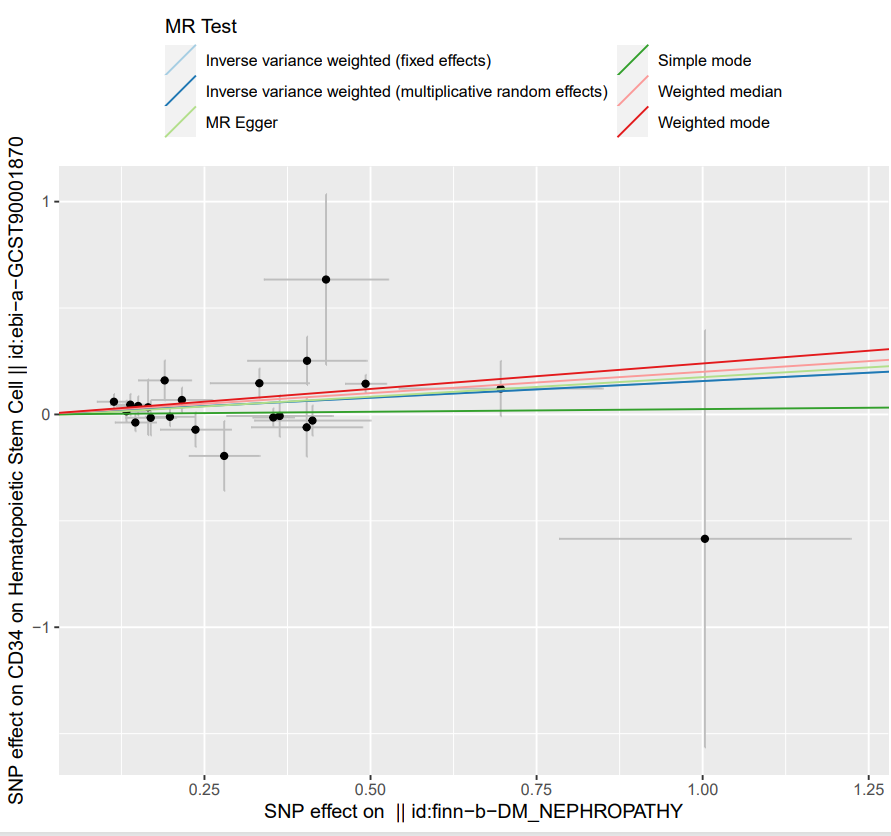

(1)Scatter plot between CD34 on Hematopoietic Stem Cell and DN risk

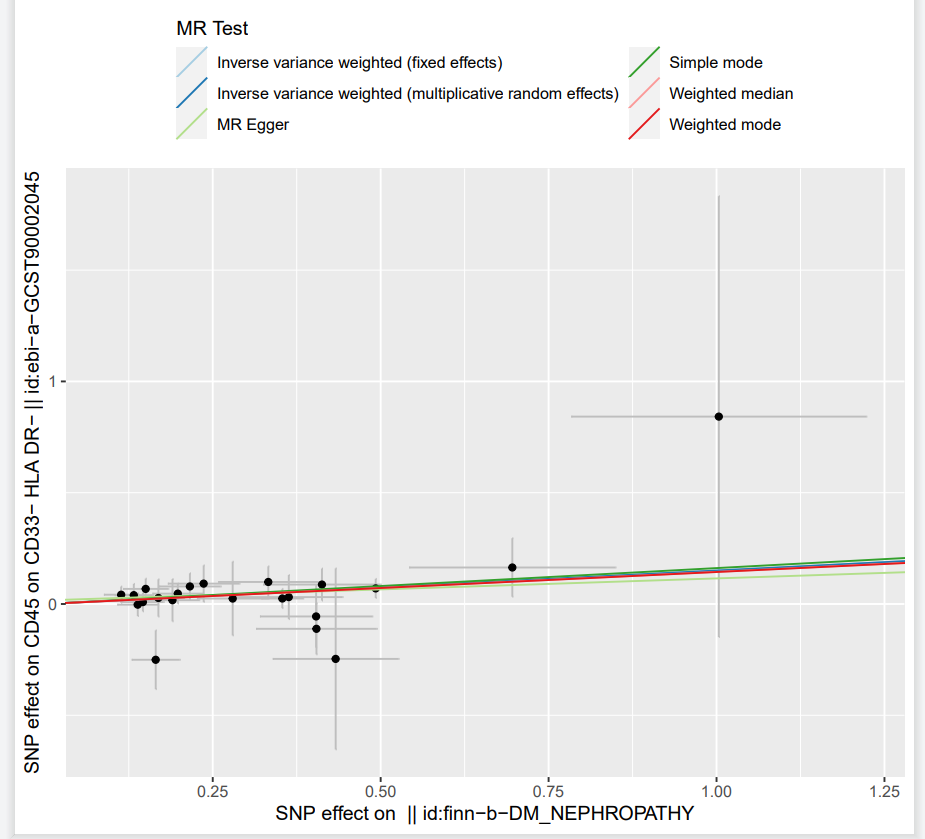

(2)Scatter plot between CD45 on CD33- HLA DR- and DN risk
