# Supplementary material for: The causal role between circulating immune cells and diabetic nephropathy: a bidirectional Mendelian randomization with mediating insights
Source: Diabetol Metab Syndr. 2024 Jul 16;16:164. doi: 10.1186/s13098-024-01386-w (PMC11253417; doi:10.1186/s13098-024-01386-w)
Supplement: Supplementary file 9 — Supplementary Material 9. Fig. S3. Causal associations between immune cells and DN. [file 13098_2024_1386_MOESM9_ESM.docx]

**Supplementary Fig. S3 Causal associations between immune cells and DN**
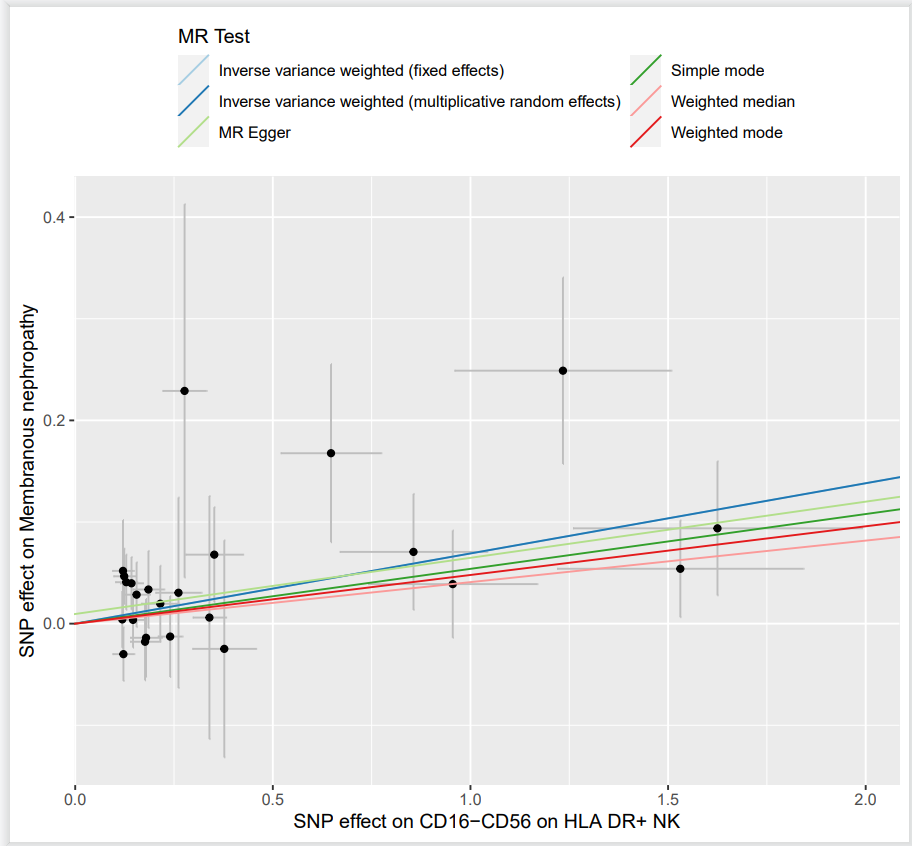

(A)Scatter plot between CD16-CD56 on HLA DR+ NK and DN risk


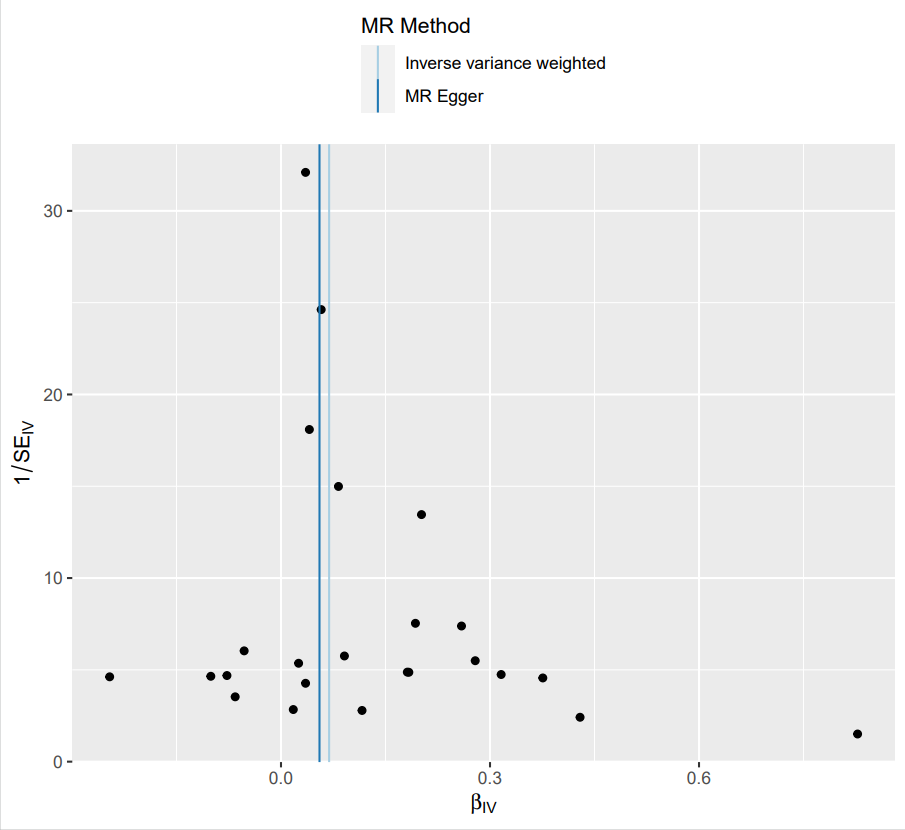

(B) Funnel plot between CD16-CD56 on HLA DR+ NK and DN risk

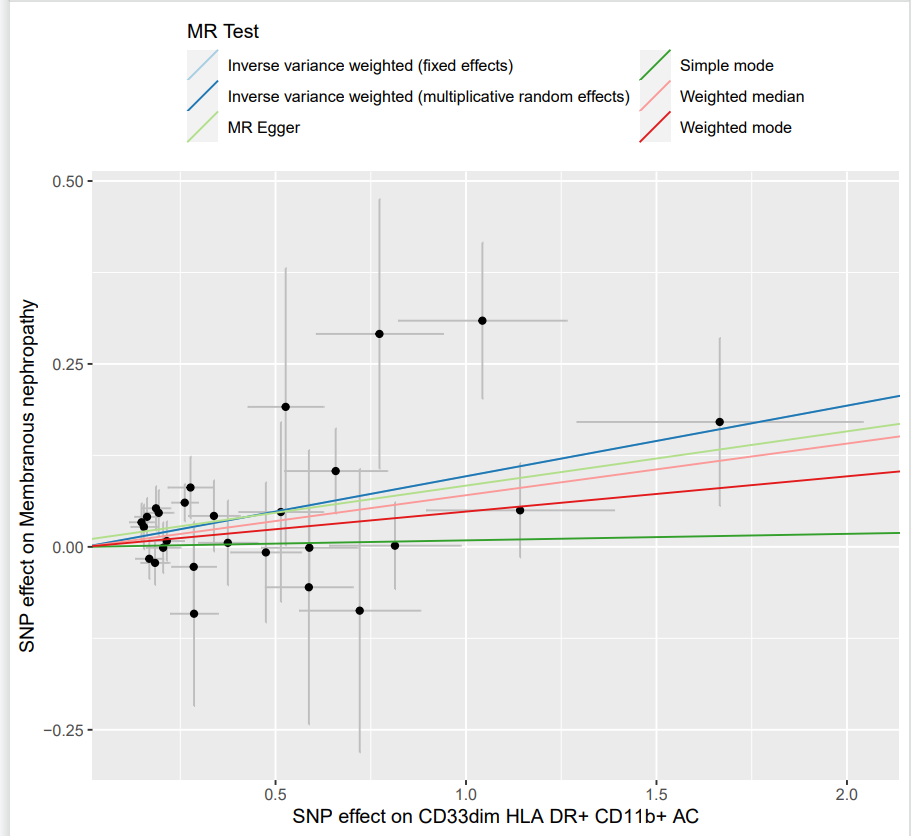

(C)Scatter plot between CD33dim HLA DR+ CD11b+ AC and DN risk

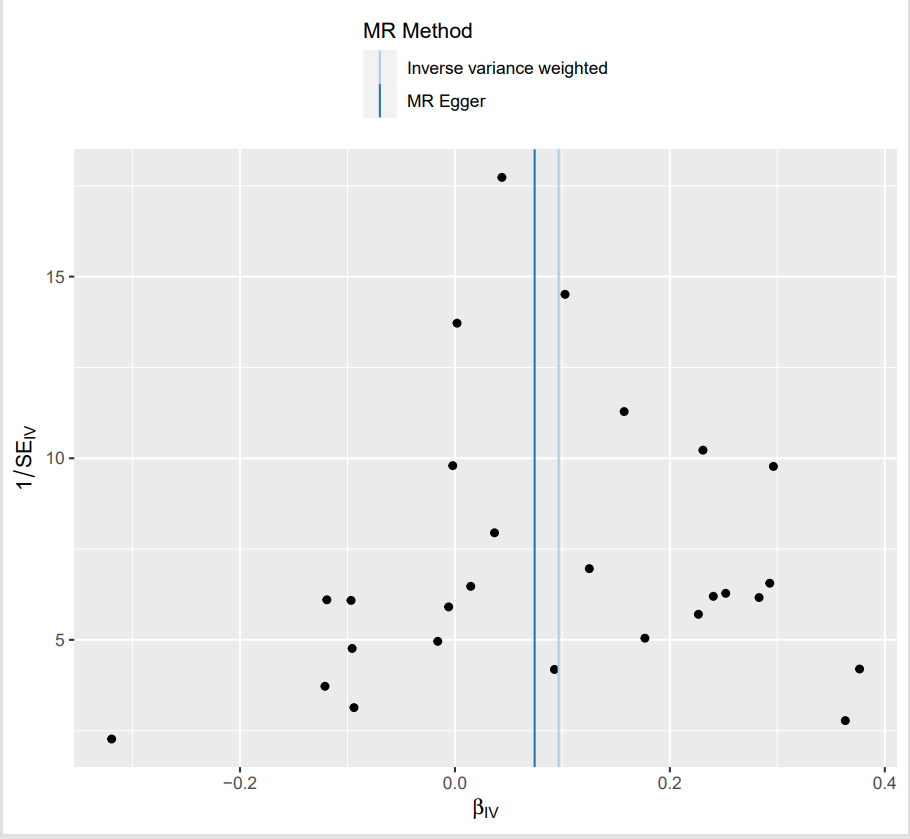

(D) Funnel plot between CD33dim HLA DR+ CD11b+ AC and DN risk


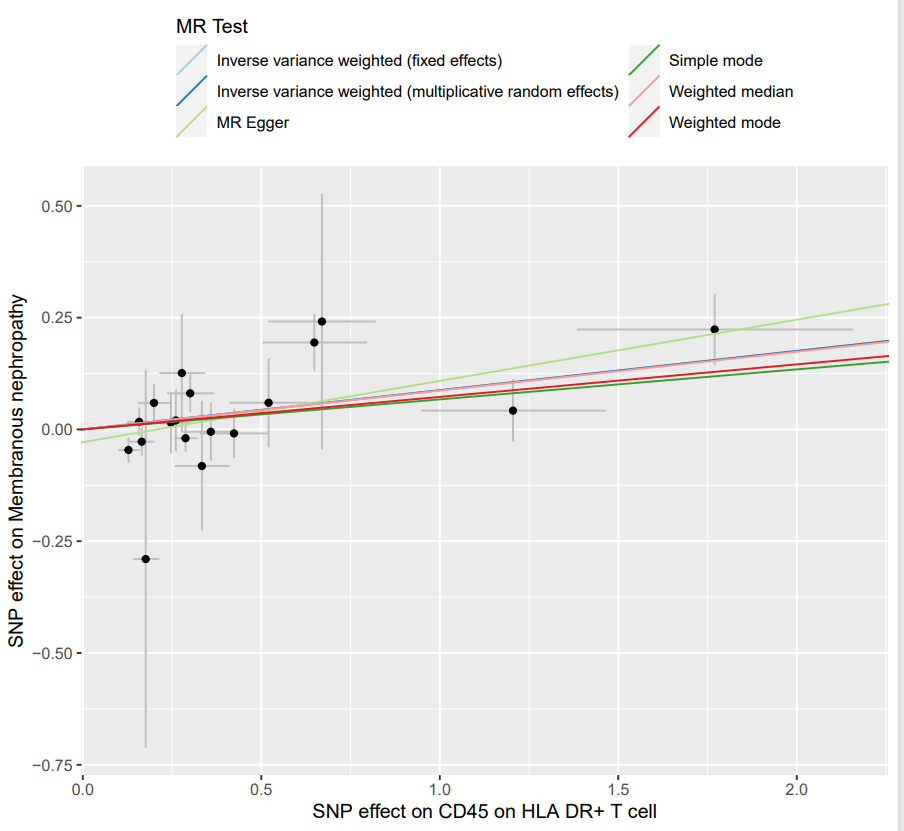

(E)Scatter plot between CD45 on HLA DR+ T cell and DN risk

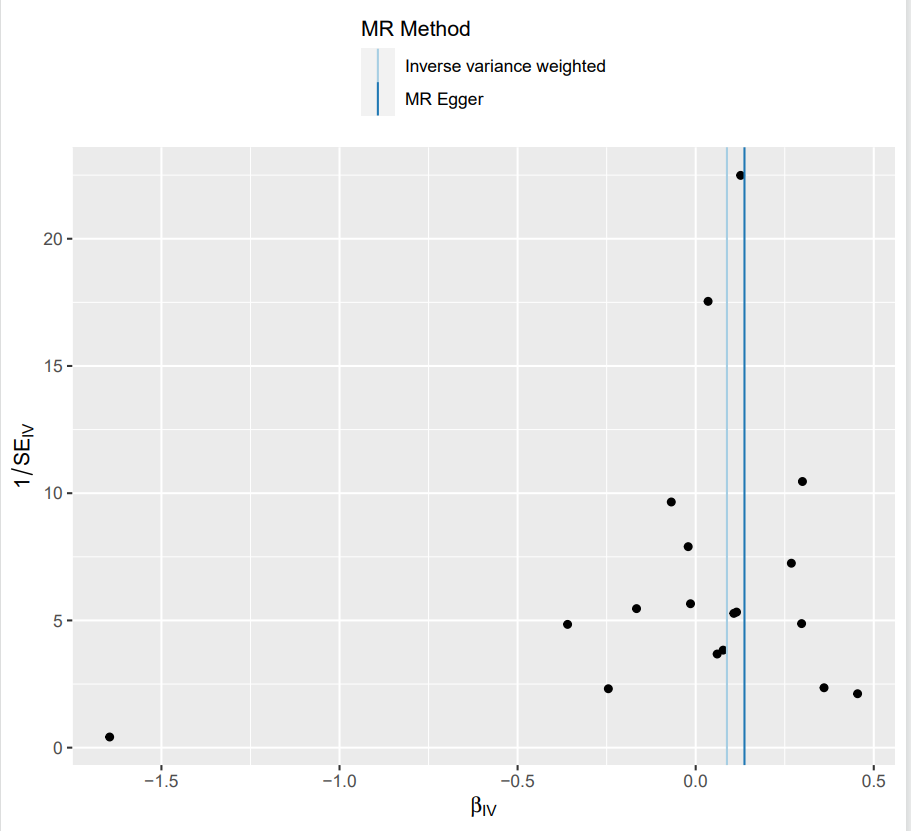

(F) Funnel plot between CD45 on HLA DR+ T cell and DN risk
